# High Spatial and Temporal Variations of Microbial Community along the Southern Catfish Gastrointestinal Tract: Insights into Dynamic Food Digestion

**DOI:** 10.3389/fmicb.2017.01531

**Published:** 2017-08-09

**Authors:** Zhimin Zhang, Dapeng Li, Mohamed M. Refaey, Weitong Xu

**Affiliations:** ^1^Department of Fishery Resources and Environment, College of Fisheries, Huazhong Agricultural University Wuhan, China; ^2^Hubei Provincial Engineering Laboratory for Pond Aquaculture Wuhan, China; ^3^Department of Animal Production, Faculty of Agriculture, Mansoura University Al-Mansoura, Egypt

**Keywords:** southern catfish, food digestion, gastrointestinal tract, microbial community, spatial and temporal variations

## Abstract

The fish intestinal microbiota is affected by dietary shifts or diet-related seasonal fluctuations making it highly variable and dynamic. It assists with the digestion and absorption of food that is a common, yet dynamic process. However, fundamental dynamics of microbial ecology associated with food digestion in intestine and stomach are poorly understood in fish. We selected the southern catfish, *Silurus meridionalis*, as the targeted species, owing to its foraging behavior with a large meal that can assure clear periodic rhythms in food digestion, to study spatial variations of the microbial community along the gastrointestinal (GI) tract. We further evaluated temporal microbial dynamics by collecting GI tract samples at time intervals 03, 12, and 24h after feeding. High-throughput sequencing results showed higher microbial diversity in the stomach than in the intestine and distinguishable community structures between stomach and intestine. *Firmicutes* were dominated by both *Clostridium* and unclassified *Clostridiaceae*, which was the most abundant taxon in the stomach, whereas *Fusobacteria* were dominated by *Cetobacterium*, which prevailed in the intestine. *Firmicutes* was significantly increased and *Fusobacteria* was decreased after feeding. Furthermore, inter-stomach microbial variability was greater than inter-intestine microbial variability. These results demonstrate that GI microbial assemblies are specific per anatomical site and are highly dynamic during food digestion, indicating that digestive status and/or sampling time are factors potentially influencing the microbial compositions. Furthermore, the finding of high spatial and temporal variations of the microbial community along the GI tract suggests limitations of single sampling regime to study food-derived microbial ecology.

## Introduction

Vertebrates harbor a wide array of symbiotic gut microbial communities that are associated with food digestion and nutrition (Wostmann, 1981; Mackie, 2002; Engel and Moran, 2013). However, gut microbial structure and composition vary dramatically among hosts even within the same host population. Gut microbiota fluctuates and shifts from days to months or years (Caporaso et al., 2011; Faith et al., 2013; David et al., 2014a). The changes in light of long-term scales can be related to individual development (Ingerslev et al., 2014; Zac Stephens et al., 2016) and seasonal variations (Keenan et al., 2013; Ye et al., 2014), providing insights into commensally host-microbiota interactions (Sugita et al., 1991; Booijink et al., 2010; Claesson et al., 2012) to elucidate microbial stability, plasticity, and evolution. Studies focusing on animals, especially for terrestrial mammals, prefer feces as a proxy of gut microbial analysis largely due to fecal sample accessibility (Crawford et al., 2009; Rolig et al., 2013; David et al., 2014a,b; Davis et al., 2016). Despite gut microbiota characterized in many vertebrates (Keenan et al., 2013; Kostic et al., 2013), most studies still have undertaken the work toward using static, rather than dynamic, status as snapshots for microbial inputs. Meanwhile, using fecal samples to estimate gut microbial community led to an unavoidable issue of whether the fecal microbiota can effectively reflect the entire gastrointestinal or regional microbiota.

Once entering the GI tract, food is subjected to differing environmental conditions along the GI tract, such as pH (Zhang Z. et al., 2016) and redox potential (Friedman et al., 2017). Decrease of mildly acidic pH significantly inhibits the growth of gut Gram-negative bacteria (Duncan et al., 2009) and leads to reduced utilization of lactate (Belenguer et al., 2007). Due to highly varying acidic milieu in stomach creating different niches potentially challenging the pH tolerance of microbiota, the community might change more rapidly in the stomach compared to that in the approximately pH-neutral intestine (Keenan et al., 2013; Beasley et al., 2015). Microbial differences between ileum and feces (Keenan et al., 2013), even between morning and afternoon, have been found in previous studies (Booijink et al., 2010). Although these studies did not consider digestive microbial dynamics in the GI tract, the results suggest that microbial composition is affected by digesta or digestive time. In addition, a marked remodeling in the microbial community of Burmese python consuming large prey at long intervals provided evidence supporting temporal variations of gut microbiota (Costello et al., 2010). It is probable that microbial assembly reflects dynamic nutrient environment in the GI tract. However, the microbial variations associated with food digestion are less well understood in animals.

Recently, although the field of fish gut microbiota has made many advances, the extent is not in parallel with the fact that fish has the largest taxonomic and ecological diversity in vertebrates (Clements et al., 2014). Unlike terrestrial mammals, fish gut microbial samples are typically collected from gut contents, mucosa or both (Ye et al., 2014; Ghanbari et al., 2015; Gajardo et al., 2016; Dehler et al., 2017). Several studies have revealed microbial differences in diverse intestinal regions such as hindgut and foregut of fish (Ye et al., 2014; Gajardo et al., 2016). Compared to the studies on intestinal microbiota, relative few existing studies based on high throughout sequencing have exploited gastric microbiota in teleosts. Furthermore, some studies controlled sampling time from hours to days since the last feeding (Sun et al., 2013; Bolnick et al., 2014; Rhodes et al., 2016), yet others did not report these details (Roeselers et al., 2011; Silva et al., 2011; Ingerslev et al., 2014; Eichmiller et al., 2016; Zac Stephens et al., 2016; Kohl et al., 2017). The scenario often occurs in field studies due to uncertainties in diet resources and randomness of feeding rhythm under natural conditions (Bolnick et al., 2014; Eichmiller et al., 2016; Llewellyn et al., 2016). Thus, it should not be overlooked to assess the microbial assemblies duiring the digestive processes. A recent overview on gut microbiota of fish highlights the importance of research planning and sampling design (Clements et al., 2014). Yet, it does not cover gut microbiota associated with dynamics of food digestion in both wild and capture fish. If sampling time and/or digestive time after feeding contributed to variations of microbial community, it could result in uncertainty of comparisons among the related studies.

Southern catfish, *Silurus meridionalis*, is an important freshwater culture species with a characteristic of rapid growth. This species is a typical representation of a stomach-containing carnivorous fish, feeding on small-sized fish (including many kinds of carps) in nature and culture ponds. The comparisons of microbiota between stomach and intestine assist in unveiling overall microbial ecology in fish GI tract. The sit-and-wait foraging tactic with a large meal in southern catfish assures clear periodic rhythms in food digestion that allows for better understanding for microbial dynamics. Thus, the purpose of this study is to compare microbial ecology between stomach and intestine in southern catfish, and further to estimate dynamic variations of GI tract microbial community after feeding. These will provide insights into the high microbial variability of GI tract in animals.

## Materials and methods

### Experimental animal and design

The experimental protocols were approved by the Animal Ethics Committee of the Huazhong Agricultural University, China, and were carried out according to the relative guidelines. A batch of 4-week-old southern catfish from a local fish farm was transported to College of Fisheries, Huazhong Agricultural University, and was reared in tanks equipped with non-circulating flow-through water system.

Prior to the experiment, healthy southern catfish were stocked in tanks (0.8 m diameter, water depth 0.36 m) for 2 weeks. During experimental period, water temperature was 26 ± 0.2°C and dissolved oxygen was 6.46 ± 0.11 mg L^−1^. The catfish were fed the same diet at a regular time (at 9:00 a.m. per day) for 6 weeks to make fish with better environmental stability including the diet, the daily feeding rhythms, and colonization of gut microbiota. The catfish was fed with pieces of crucian carp (*Carassius carassius*) without the head and viscera. After half an hour of feeding, uneaten food was immediately removed from the tanks. At the end of the experiment, eight fish were collected randomly from two tanks (four fish per tank) at 03, 12, and 24h after feeding, respectively. Sampling at 24h after feeding occurred before the next feeding moment. After fish were anesthetized with MS-222, fish body weight and length of the fish were measured (Table S1), and the stomach and lower half of the intestine were aseptically removed. Intestinal contents were squeezed into a sterile tube. Similarly, the contents of stomach were collected. However, we did not collect the stomach samples at 24h after feeding because no food, besides occasionally some observed fishbones, was found in the stomach. The intestine samples collected at 03, 12, and 24h after feeding were named of Int:03h, Int:12h, and Int:24h, meanwhile the stomach samples at 03 and 12h were named of Sto:03h and Sto:12h, respectively. The contents of stomach and intestine at the different intervals time are shown in Figure S1. Each sample was homogenized and immediately stored at −80°C until microbial analysis.

### Measurements of gastrointestinal tract pH

Small slits introduced in the GI tract were prepared for measurements of gastrointestinal pH *in vivo*. The GI tract pH was detected with three replicates per sample using a specialized pH meter (Testo 205, Testo, Germany) by directly inserting the electrodes of pH meter through the GI epithelium into the lumen.

### DNA extraction, PCR and sequencing

Genomic DNA was extracted from 16 stomach samples and 24 intestine samples using the QIAamp DNA Stool Mini Kit (Qiagen, Hilden, NRW, Germany) following the manufacturer's protocol. The V4-V5 hypervariable region of bacterial 16S rRNA gene was amplified using universal primers 515F (5′-GTGCCAGCMGCCGCGGTAA-3′) and 907R (5′-CCGTCAATTCCTTTGAGTTT-3′). The 515F primers were designed to include at the 5′-end a unique index tag barcode of 12 bases allowing identifications of different samples. PCR mixtures contained 0.5 μM of each forward and reverse primer, 100 ng of template DNA, 2.5U of GoTaq Flexi Polymerase (Promega, Madison, WI, USA), 200 μM of dNTPs, and 2 mM of MgCl_2_ in a final volume of 50 μl. The PCRs were performed in a Biorad T100 (Biorad, Hercules, CA,USA) with an initial denaturation step at 94°C for 5 min, followed by 25 cycles of 94°C for 30 s, 55°C for 30 s, 72°C for 1 min and a final extension 72°C for 5 min. We visualized the PCR products on a 1% agarose gel. The target products (~400 bp) were cut and purified using the Qiagen Gel Extraction Kit, and then were quantified using the Nanodrop 2000 Spectrophotometer (ThermoFisher, Waltham, MA, USA). After the individual quantification step, amplicons were pooled in equal amounts and the pool was used to prepare the Illumina sequencing library using the TruSeq DNA kit according to the manufacturer's instruction. The sequencing was performed on an Illumina HiSeq 2500 sequencing platform with the PE250 sequencing strategy according to the manufacturer's instruction. Raw data were deposited to the NCBI BioProject under accession number PRJNA373828.

### Sequence processing and statistical analysis

The raw sequence data were processed using QIIME Pipeline-Version 1.7.0 (http://qiime.org/tutorials/tutorial.html). All sequences were trimmed and assigned to each sample based on their barcodes (barcode mismatches = 0). The overlapping paired-end reads were merged using the FLASH-1.2.8 software (Magoč and Salzberg, 2011). The merged sequences with high quality (read length >300 bp, without ambiguous base “N,” and average base quality score >30) were used for further analysis. All sequence reads were sorted based on their unique barcodes. Chimera sequences were removed using the UCHIME algorithm (Edgar et al., 2011). Sequences were then resampled to the same sequence depth (22000 reads per sample except for one stomach sample with less sequence data) using daisychopper.pl (http://www.festinalente.me/bioinf/downloads/daisychopper.pl) for downstream analysis. These sequences were clustered into operational taxonomic units (OTUs) at 97% sequence identity cut-off using UCLUST algorithm and singletons were filtered out. Ribosomal Database Project (RDP) classifier was used for the taxonomic assignment.

Student's *t*-test was used to estimate differences in alpha diversity (observed species and Phylogenetic diversity[PD] whole tree) between stomach and intestine, and those in the stomach between sampling time points, meanwhile one-way analysis of variance (ANOVA) was used to examine the differences in intestine among different time points using SPSS 20.0. We performed non-metric multidimensional scaling analysis (NMDS) for GI microbial community at OTU level with Bray-Curtis distance. And a hierarchical clustering was built based on Bray-Curtis distance among groups using R (http://www.r-project.org/). Unweighted and weighted UniFrac phylogenetic distance metrics were used with principal coordinate analysis (PCoA) to further visualize variations of community members and structure. To explore the variability of GI microbial community during the digestion, we determined both Bray-Curtis and weighted UniFrac distances of samples within groups and between groups.

We performed PERMANOVA analysis for stomach and intestine microbial community as well as pair-wise comparisons of PERMANOVA analysis on weighted UniFrac distance for microbial structure. Furthermore, the dissimilarity analysis of the microbial community structure at the phyla levels between groups was evaluated and the contributions of specific taxon to the dissimilarity were calculated by the similarity percentage analysis (SIMPER) in Past 2.0. Mann-Whitney U-test was used for comparisons of two groups, whereas Kruskal-Wallis test was used for comparisons of more groups in term of the relative abundance of a taxonomic composition. The differences in the pH of GI tract different time points were analyzed by one-way ANOVA. All statistical significance in this study was set at a *P*-value < 0.05.

### Microbial function prediction

The microbial functionality profiles associated with each sample were predicted using Phylogenetic Investigation of Communities by Reconstruction of Unobserved States (PICRUSt) to generate the Kyoto Encyclopedia of Genes and Genomes (KEGG) pathway. The sequences were normalized by subsampling for functional characterization to minimize differences in 16S rDNA copy number that were mapped to Greengenes ver. 13.5 database for functional prediction. The predicted genes and their function were aligned to KEGG database and the differences among groups were compared using STAMP (http://kiwi.cs.dal.ca/Software/STAMP). Two-side Welch's *t*-test and Benjamimi-Hochberg FDR correction were employed for comparisons of two groups.

## Results

### Microbial alpha diversity of gastrointestinal tract

**Figure 1:**
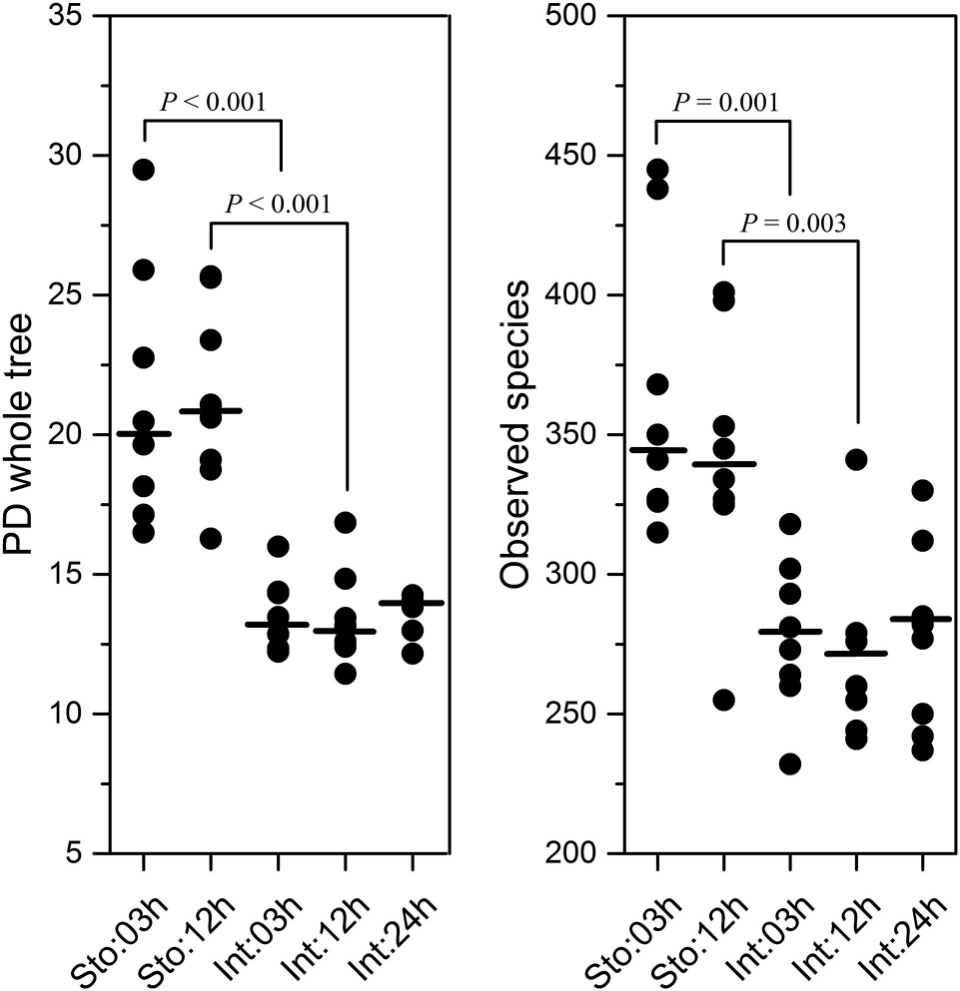
Diversity and species richness estimation of stomach and intestine microbiota of southern catfish after feeding. Phylogenetic diversity (PD) whole tree and observed species measurements calculated after rarifying samples to equal sequencing depth in QIIME.

After rarefaction, quality and criteria filtering of raw reads, a total of 3,589,904 high-quality sequences were obtained from 40 GI tract samples collected at time intervals 03, 12, and 24h after feeding. Total 1619 OTUs from the GI tract were generated with 864 OTUs shared by stomach and intestine, with 476 and 279 unique OTUs, respectively (Figure S2). The 275 bacterial OTUs were shared among all sampling time-points. More OTUs are unique to the stomach (Sto:03h = 124 and Sto:12h = 138) compared to the intestine (Int:03h = 28, Int:12h = 28 and Int:24h = 38) (Figure S2). The stomach had higher microbial diversity at 03h (Student's *t*-test, *P* < 0.001 for both PD and *P* = 0.001 for observed species) and at 12h (*P* < 0.001 for PD and *P* = 0.003 for observed species) than the intestine (Figure 1). Regardless of sampling time, similar results were observed (*P* < 0.001 for both PD and observed species). However, there was no difference in the microbial diversity at 03 and 12h for the stomach (Student's *t*-test, *P* = 0.979 for PD; *P* = 0.390 for observed species) and among the three time points for the intestine (one-way ANOVA, *P* = 0.942 for PD; *P* = 0.916 for observed species).

### The differences of microbial communities between the stomach and intestine

**Figure 2:**
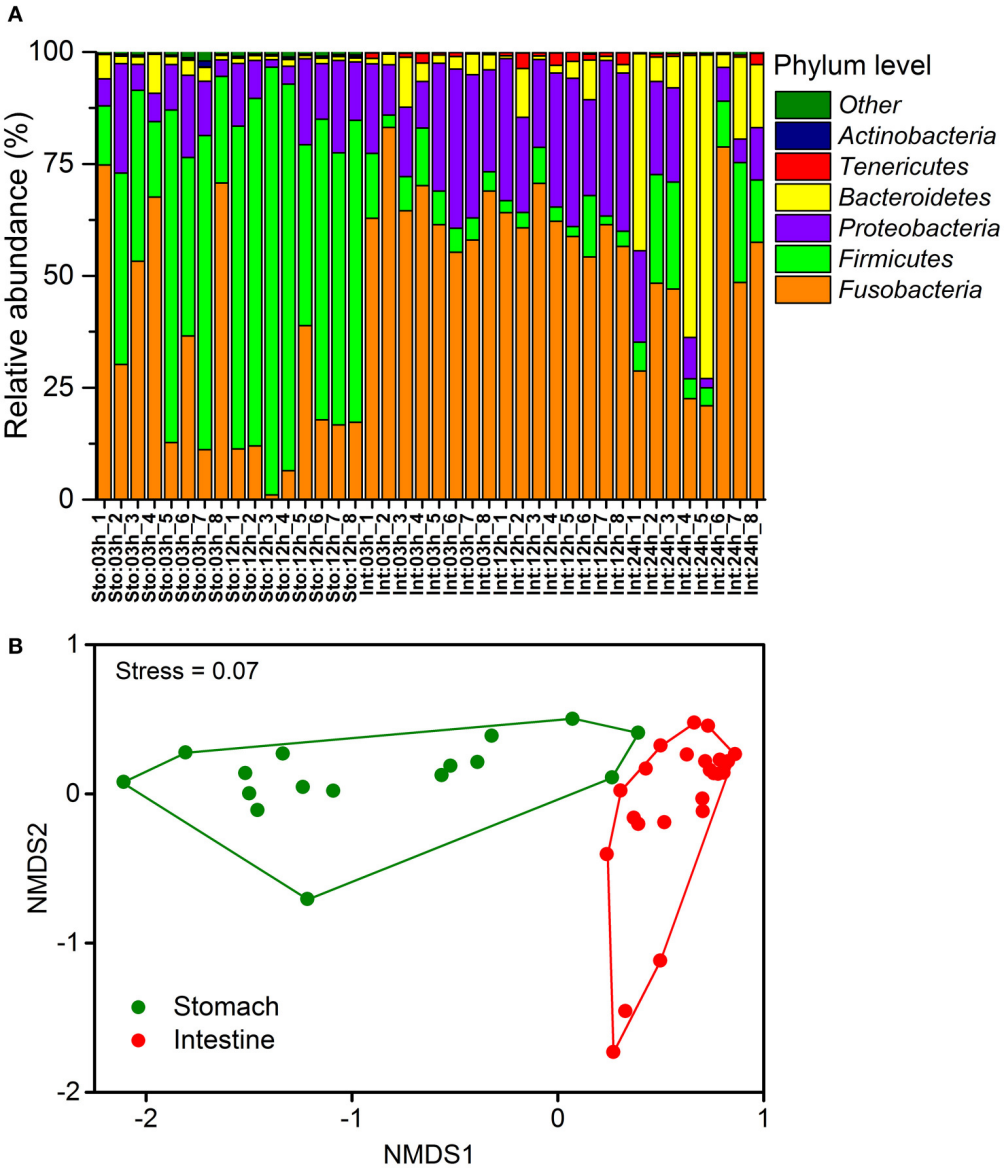
Temporal dynamics in microbial community compositions of the gastrointestinal tract of southern catfish after feeding. **(A)** Relative abundance of the phyla of all samples at 03, 12, and 24h after feeding; **(B)** Non-metric multidimensional scaling ordinance based on a distance matrix computed with Bray-Curtis distance.

The most abundant phyla across all samples were *Fusobacteria, Firmicutes, Proteobacteria*, and *Bacteroidetes* (Figure 2A). Each of these phyla showed significant differences in relative abundance between the stomach and intestinal samples (on average 29.9 and 56.9% for *Fusobacteria*, 55.4 and 8.9% for *Firmicutes*, 11.3 and 20.9% for *Proteobacteria* and 21.9 and 12% for *Bacteroidetes*). The differences were also observed in the less abundant phyla (such as 0.2 and 1.1% for *Tenericutes*, 0.3 and 0.002% for *Actinobacteria* in the stomach and intestine, respectively). The phylum *Fusobacteria* in the stomach and intestine were dominated by the genus *Cetobacterium*, the *Firmicutes* by unclassified *Clostridiaceae, Clostridium*, and *Bacillus*, and the *Proteobacteria* by the *Plesiomonas* (Table S2). The SIMPER revealed overall dissimilarity (52.93%) between stomach and intestine (Table S3). The contributions of *Firmicutes* and *Fusobacteria* to the dissimilarity were 44.32 and 31.81%, respectively. We found dramatic differences in microbial composition of the stomach and intestine at the OTU levels (Figure S3) that were visualized by a non-metric multidimensional scaling (NMDS) plot based on Bray-Curtis distance (Figure 2B). Moreover, there were substantial effects of organs for shaping differences in community structure (Bray-Curtis, one-way PERMANOVA, *P* = 0.0001).

### Temporal dynamics of microbial communities

**Figure 3:**
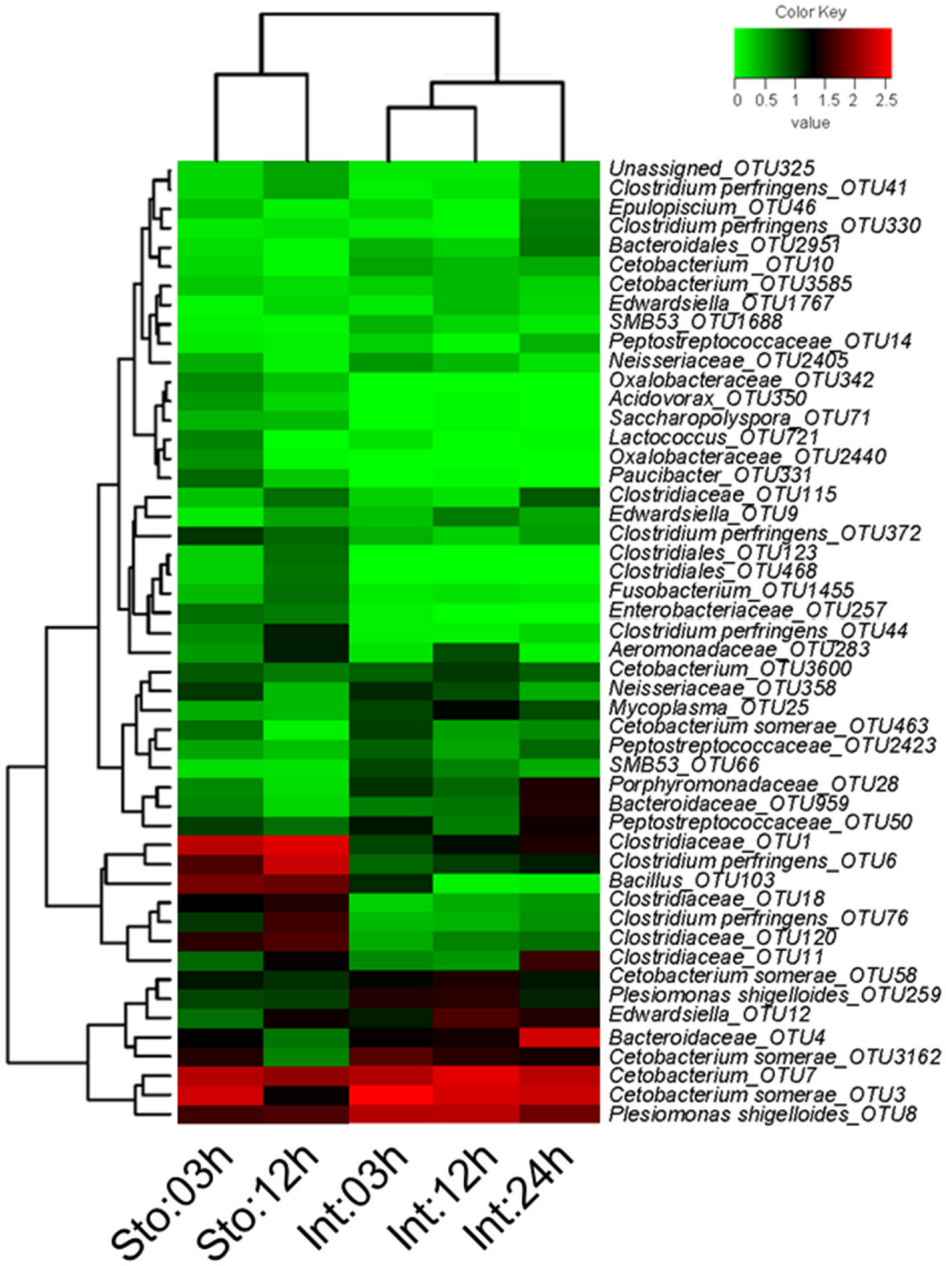
Heatmap of showing the relative abundance reveals obvious microbial dynamics and compositional differences between stomach and intestine after feeding. Columns are arranged by similarity using hierarchical clustering. The relative abundance data was log 10 transformation. Bray-Curtis clustering based on top 50 OTUs in the stomach and intestine.

We determined postprandial variations of bacterial communities over time and found temporal microbial dynamics (Figure 3). Statistical analyses revealed that sampling time resulted in variable community structures (Bray-Curtis, one-way PERMANOVA, *P* = 0.005 for stomach and *P* = 0.0007 for intestine). PCoA based on UniFrac distance revealed separations in the microbial community of GI tract among sampling time points (Figure S4). These temporal differences were mostly found in the weighted UniFrac distance, suggesting that these communities differ in terms of relative abundance, not in the presence/absence of certain taxa. The unweighted UniFrac distance did not show clear time point clustering within a sample type. The pair-wise comparisons further revealed significant differences in the microbial structure at different time points (Table 1).

**Table 1:** Pair-wise comparison of microibota at OTU level of the gastrointestinal tract at different time points after feeding^#^.

**Groups**	**Int:03h**	**Int:12h**	**Int:24h**	**Sto:03h**
Int:12h	0.0013			
Int:24h	0.0126	0.0196		
Sto:03h	0.0005	0.0007	0.0402	
Sto:12h	0.0001	0.0004	0.0004	0.0042

# *The comparison was performed using PERMONOVA on weighted UniFrac distance*.

**Figure 4:**
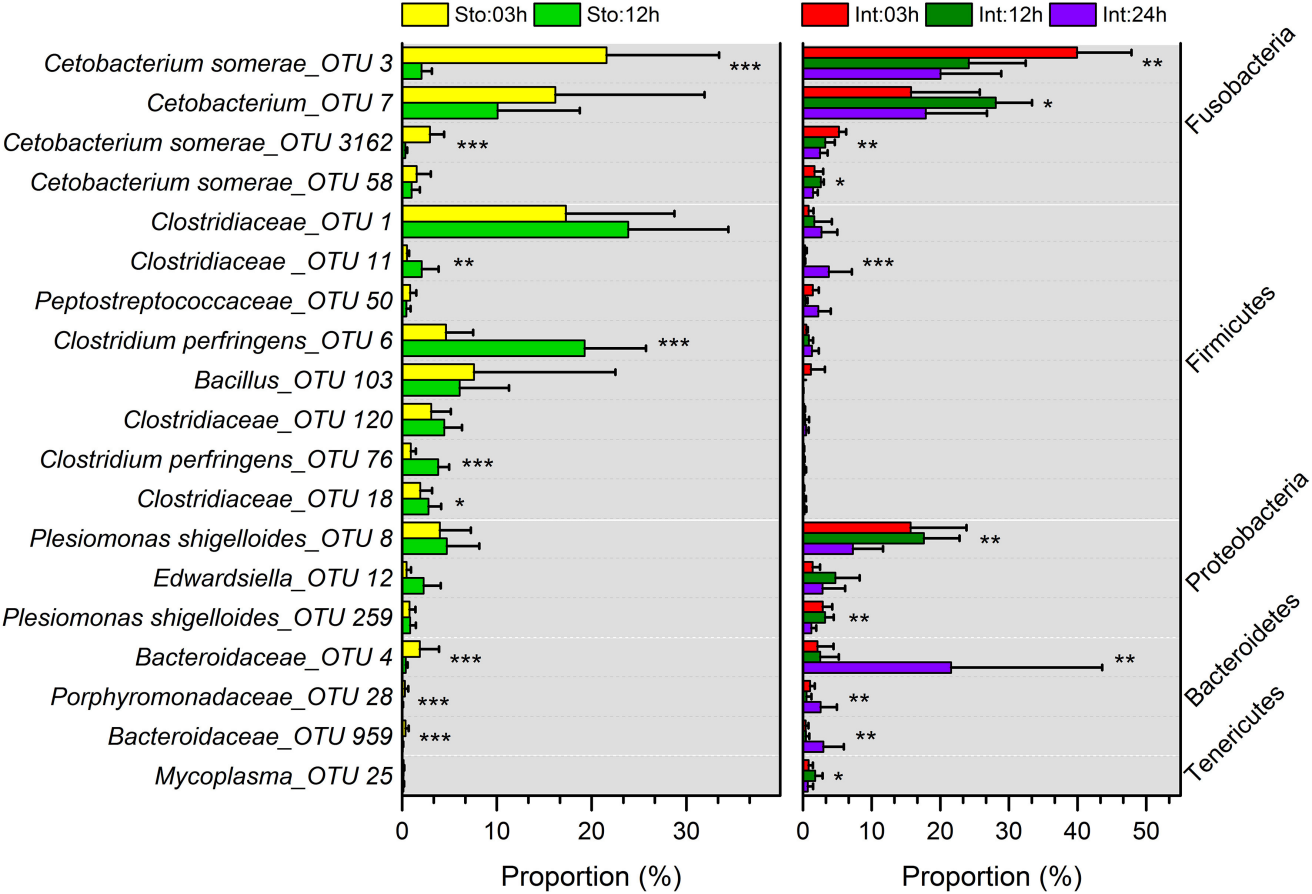
Comparisons of microbial taxonomic compositions of southern catfish gastrointestinal tract at different time points after feeding. The relative abundances (> 0.5%) at the OTU levels in stomach and intestine are presented. Asterisks indicate significant differences (^*^P < 0.05, ^**^P < 0.01, ^***^P < 0.001).

Stomach community at 03h after feeding was clearly divided from that at 12h by the lower abundance of *Firmicutes* (on average, 39.91 vs. 70.95%) and higher *Fusobacteria* (44.62 vs. 15.18%) (Figure S5A). The phyla were the two largest contributors (44.25 and 41.68%) to the overall dissimilarity (38.98%) (SIMPER, Table S3). The intestine microbiota at 03 and 12h after feeding had less overall dissimilarity (13.87%), which increased between groups over time (Table S3). The abundance of *Fusobacteria* and *Proteobacteria* significantly decreased at 24h after feeding (Figure S5A). Of note, *Bacteroidetes* in the intestine at 03h dramatically increased from 3.94 to 28.38% at 24h after feeding, resulting in the largest contribution (34.66%) to the overall dissimilarity (SIMPER, Table S3). Similarly, conspicuous differences were also detectable at genus levels (Figure S5B). The genus *Cetobacterium* significantly decreased from 44.48% at 03h to 14.69% at 12h after feeding in the stomach (Student's *t*-test, *P* = 0.027), and from 65.51% at 03h to 44.02% at 24h in the intestine (one-way ANOVA, *P* = 0.014), whereas unclassified *Clostridiaceae* increased from 23.03 to 35.76% in the stomach (Student's *t*-test, *P* = 0.027) and from 1.5 to 7.84% in the intestine (one-way ANOVA, *P* = 0.016) (Table S2). *Clostridium* increased from 7.08 to 26.55% in the stomach (Student's *t*-test, *P* = 0.001) and unclassified *Bacteroidaceae* decreased from 2.63 to 25.18% in the intestine during digestion (one-way ANOVA, *P* = 0.007) (Table S2). In addition, most dominant OTUs in the stomach and intestine changed significantly after feeding (Figure 4).

### Inter-individual variability of gastrointestinal microbiota

**Figure 5:**
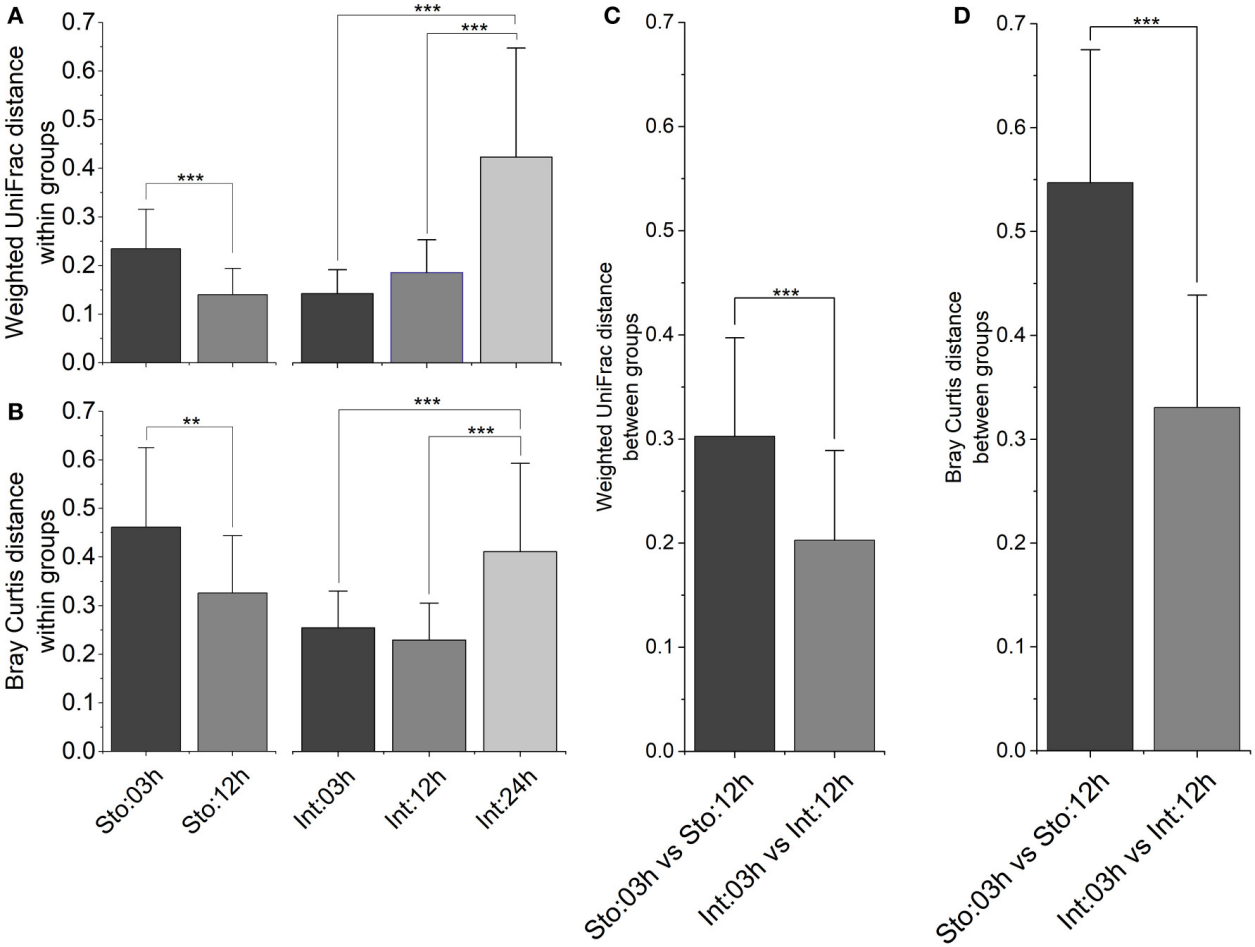
Inter-individual variations of the gastrointestinal microbiota after feeding. Bar plot of mean and standard deviation within groups calculated by **(A)** weighted UniFrac distance and **(B)** Bray-Curtis distance, and between groups calculated by **(C)** weighted UniFrac distance and **(D)** Bray-Curtis distance. Asterisks indicate significant differences (^*^P < 0.05, ^**^P < 0.01, ^***^P < 0.001).

To determine whether microbial community among samples within time points has different variability after feeding, and whether sample type modulates the different processes for microbial assemblages, several different distance metrics were used to assess variations of GI tract microbiota community. In the stomach, the average within-group weighted UniFrac distances (Figure 5A, *P* < 0.001) and Bray-Curtis distances (Figure 5B, *P* < 0.01) were significantly lower at 12h than at 03h after feeding. In contrast, there were no significant differences in the intestine at 03 and 12h. However, both distances for the intestine at 24h increased robustly compared to those at 03 and 12h after feeding (Figures 5A,B, *P* < 0.001 for both). We further visualized how similar was GI microbiota of individuals between 03 and 12h after feeding time points. The results showed higher individual distances in the stomach than the intestine for both distance metrics (Figures 5C,D, *P* < 0.001 for both), suggesting larger fluctuations in microbial community structure in the stomach compared to the intestine during the digestive process.

### pH changes in gastrointestinal tract after feeding

**Figure 6:**
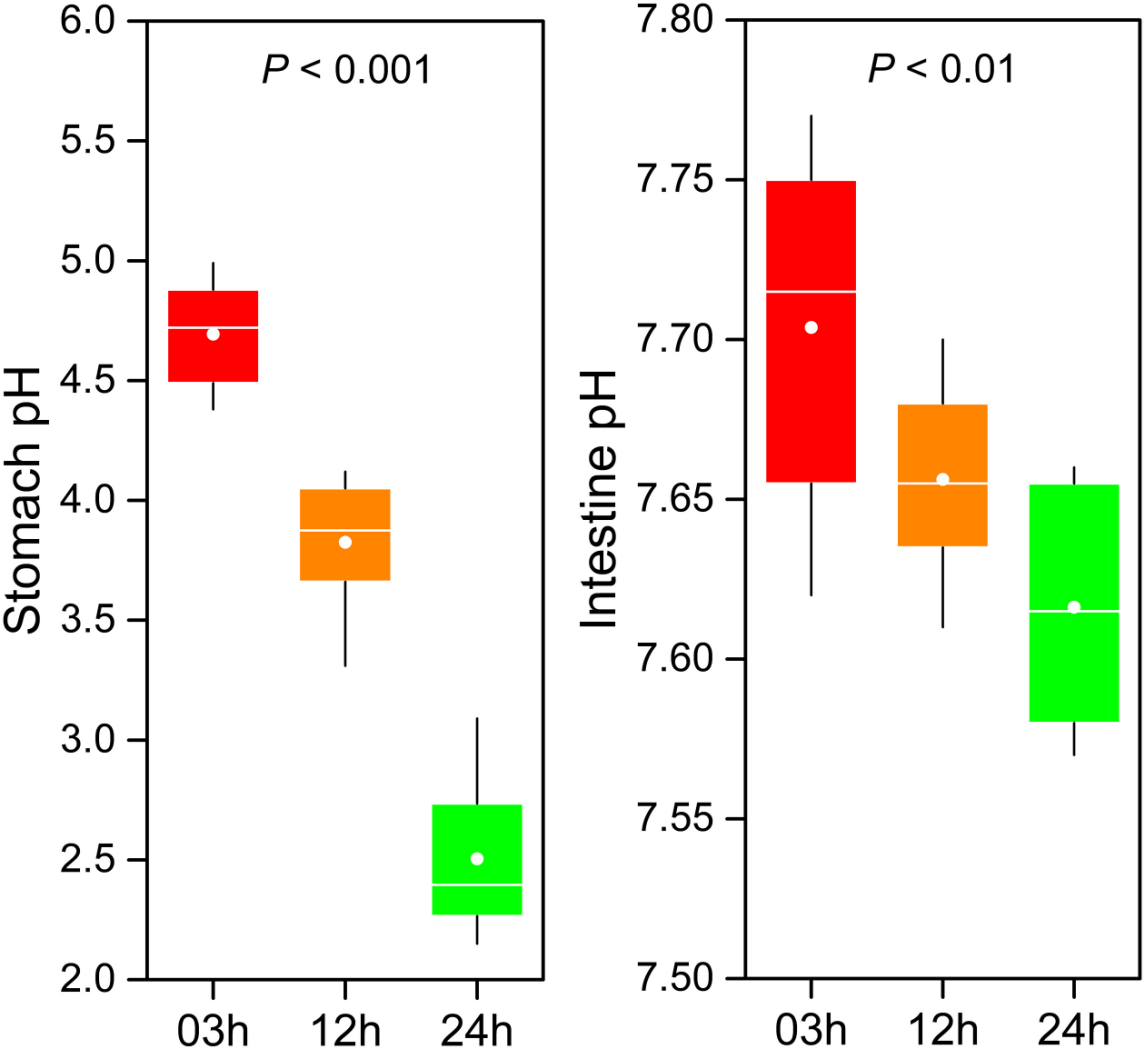
pH change in the gastrointestinal tract of southern catfish after feeding.

The GI tract environment changes with food digestion. During digestion, the pH of GI tract (including the stomach and intestine) significantly decreased over time (Figure 6). A significantly lower stomach pH was observed compared to intestine pH. On average, pH in the stomach ranged from 4.69 at 03h to 2.5 at 24h after feeding (one-way ANOVA, *P* < 0.001). Although pH in the intestine was subjected to relatively small changes (from 7.7 to 7.62) during digestion, the difference was statistically significant (one-way ANOVA, *P* < 0.01).

### Functional prediction using PICRUSt

**Figure 7:**
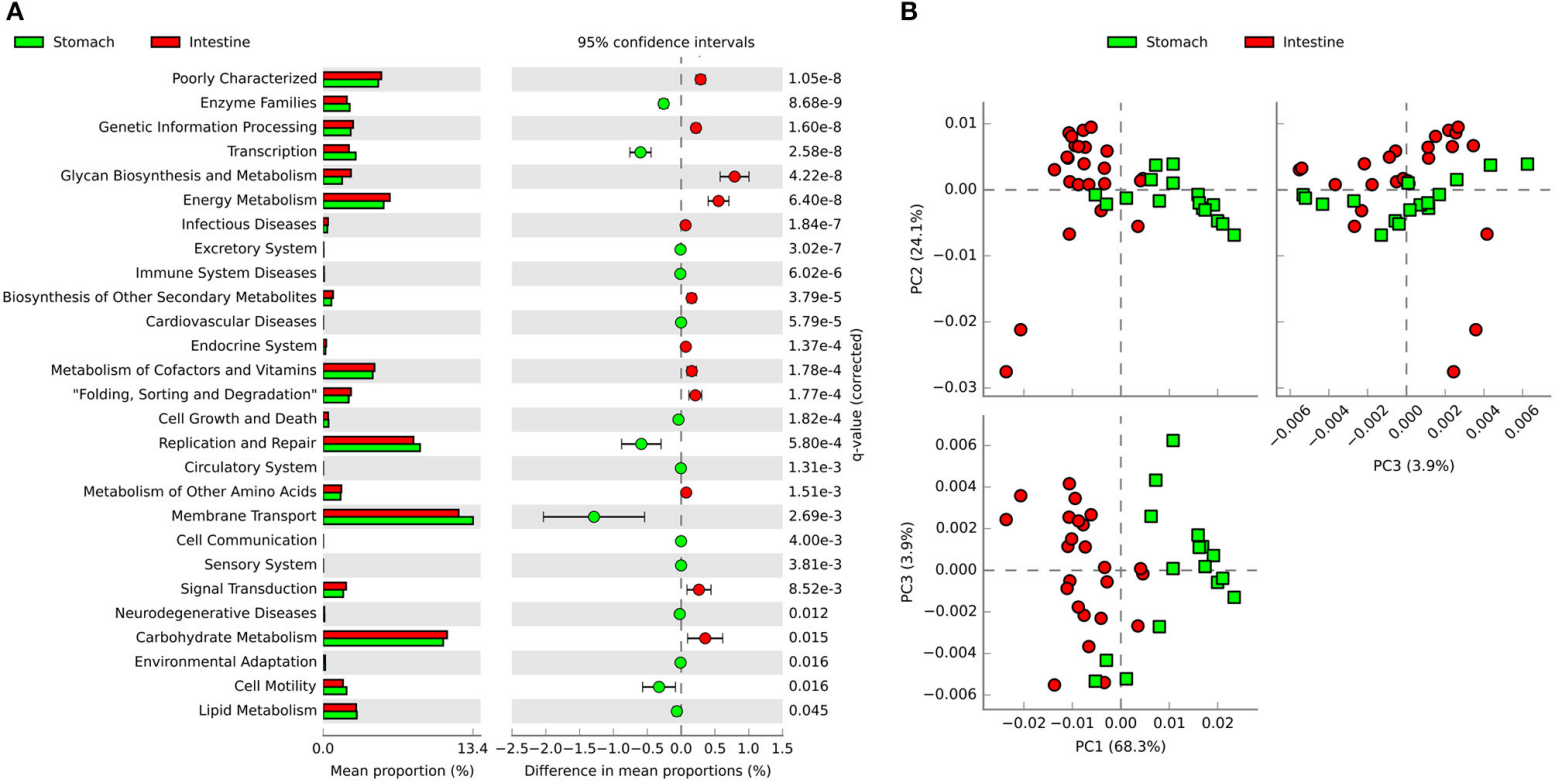
PICRUSt classification of KEGG Orthologies (KO) in the gastrointestinal tract of southern catfish. **(A)** Mean proportion and the differences in predicted functional genes of the gastrointestinal tract microbiota at KEGG level 2; **(B)** Principal components analysis (PCA) of predicted functional genes of gastrointestinal tract microbiota at KEGG level 3.

PICRUSt was used to predict functional genes of microbial communities in southern catfish GI tract. Using the level 2 KEGG ortholog function predictions, we found 27 significantly different functional categories between the stomach and intestine (Figure 7A). The functional categories associated with microbiota in the intestine compared to those in the stomach included notable enrichment of several metabolic pathways, such as energy metabolism, glycan biosynthesis and metabolism, carbohydrate metabolism, and metabolism of cofactors and vitamins, whereas the abundance of functional genes in enzyme families, transcription, membrane transport, and replication and repair was significantly lower in the intestine. At the KEGG level 3, principal components analysis (PCA) based on the abundance of functional genes of microbial communities in the GI tract showed clear separations between stomach and intestine samples (Figure 7B), indicating functional differences in microbial communities between the stomach and the intestine. We identified numerous significantly enriched functional pathways such as transporters, peptidases, transcription factors and ABC transporters in the stomach compared to the intestine (Figure S6). In addition, the predicted functions of the GI tract microbiota also showed temporal differences during the digestion (Figure S7).

## Discussion

Exploring the gaps and dynamics of microbial community among GI tract and the effects of environmental factors on microbial assemblies contributes to comprehensive insights into host microbial ecology (Brüssow, 2016; Laukens et al., 2016). In this study, we found significant differences in microbial community between the stomach and intestine of southern catfish, reflecting divergent microbial ecology of specific GI tract habitats. Moreover, we defined the postprandial variability of GI microbiota after feeding. These findings indicate divergences between microbial consortia, highlighting the importance of habitat ecology for microbial colonization in the GI tract and the necessity of controlling for temporal variability of comparative studies of GI microbiota.

Selection in the host determines the gut microbiota assembly and colonization success. Despite the existence of *Fusobacteria* in fish GI tract, *Proteobacteria* dominates the intestine of many fish species with microbial differences (Roeselers et al., 2011; Xia et al., 2014). However, *Fusobacteria* was the most abundant in the GI tract of southern catfish. Furthermore, we integrated studies reporting the dominance of *Fusobacteria* in fish (Table 2). In addition to freshwater habitats, we found no common characterizations for these fish in feeding habits, diet categories, sample origins, and sequencing techniques. The species-level taxonomy *Cetobacterium somerae* belonging to *Fusobacteria* mainly assigned to OTU 3 in this study thrived in the GI tract of the freshwater fish. However, a meta-analysis of 25 fish species with varying feeding habits and habitats displayed low abundances (only 2.88% of the mean prevalence) of *Fusobacteria* (Sullam et al., 2012). Although feeding preferences were used to explain significant differences, especially for wild populations, in gut microbial communities of animals (Miyake et al., 2015; Liu et al., 2016), captive populations (several carp fish species) with higher *Fusobacteria* abundance were more similar than the corresponding wild counterparts (Eichmiller et al., 2016) and significantly differed from other populations (Li et al., 2012, 2014; Ye et al., 2014). One possible explanation for the differences may be associated with the intake of vitamin B_12_ from the diet as it has been reported that *C*. *somerae* has vitamin B_12_-producing ability in the GI tract of freshwater fish (Sugita et al., 1991; Tsuchiya et al., 2008). GI microbial assemblies are the reflections of environmental and certain specific host physiological stress (Sun et al., 2013) linking to potential metabolic modulations of GI microbiota and in turn metabolites, such as vitamin B_12_,as modulators of gut microbial ecology (Degnan et al., 2014).

**Table 2:** Bacteria assigned to the phylum *Fusobacteria* are abundant in the gastrointestinal tract of different freshwater fish species.

**Fish species**					**Target/techniques**	**Phylum**	**Genus**	**Species**	
**English name**	**Latin name**	**FH**	**Fish origins**	**Sample origins**		***Fusobacteria***	***Cetobacterium***	***C. somerae***	**References**
Cichlid Fishes^$^	*Haplotaxodon microlepis, Haplotaxodon trifasciatus, Plecodus straeleni, Perissodus microlepis* and *Perissodus eccentricus*.	Z, O, and C	Wild	Intestinal tissues	16S/454 pyroseqeuncing, V1-V2 and V3-V4	~40%	/	/	Baldo et al., 2015
*Panaque* catfishes^$^	*Panaque sp*.^#^	O	Captivity (Pellet)	Faeces (externally)	16S/454 pyroseqeuncing,	/	72.90%	/	Di Maiuta et al., 2013
					V1-V3				
*Panaque* catfishes^$^	*Panaque sp*.^#^	O	Captivity (Wood)	Faeces (externally)	16S/454 pyroseqeuncing,	/	74.70%	/	
					V1-V3				
Siberian sturgeon	*Acipenser baerii*	C	Wild	Hindgut contents	16S/454 pyroseqeuncing, V3	dominance	/	51.14%	Geraylou et al., 2013
Bluegill	*Lepomis macrochirus*	O	Pond^§^	Intestinal contents	16S/454 sequencing	/	82.60%	/	Larsen et al., 2014
Largemouth black bass	*Micropterus salmoides*	C	Pond^§^	Intestinal contents	16S/454 sequencing	/	90.56%	/	
Channel catfish	*Ictalurus punctatus*	O	Pond^§^	Intestinal contents	16S/454 sequencing	/	94.13%	/	
Grass carp	*Ctenopharyngodon idellus*	H	Aquaculture and wild	Intestinal mucosa and contents	16S/DGGE + Sanger sequencing, V3	/	dominance	/	Ni et al., 2012
Southern catfish	*Silurus meridionalis*	C	Lab	Gastric contents	16S/HiSeq 2500, V4-V5	29.90%	29.59%	15.43%	In this study
Southern catfish	*Silurus meridionalis*	C	Lab	Intestinal contents	16S/HiSeq 2500, V4-V5	56.90%	56.86%	34.98%	
Common carp	*Cyprinus carpio*	O	Lab (Pellet)	Faeces (internally)	16S/HiSeq 2000, V6	~50%	/	/	Eichmiller et al., 2016
Freshwater drum	*Aplodinotus grunniens*	C	Wild	Faeces (internally)	16S/HiSeq 2000, V6	~40%	/	/	
Common carp	*Cyprinus carpio*	O	Lab (Brine shrimp)	Faeces (internally)	16S/HiSeq 2000, V6	~40%	/	/	
Crucian carp	*Carassius auratus*	O	Lab (Flake food)	Faeces (internally)	16S/HiSeq 2000, V6	~40%	/	/	
Bighead carp	*Hypopthalmichthys nobilis*	Z	Lab (Algal feed mixture)	Faeces (internally)	16S/HiSeq 2000, V6	~35%	/	/	
Yellow catfish	*Pelteobagrus fulvidraco*	O	Wild	Intestinal contents	16S full length/Sanger sequencing	27.00%	/	/	Wu et al., 2010
Yellow catfish	*Pelteobagrus fulvidraco*	O	Wild	Intestinal mucosa	16S full length/Sanger sequencing	31.60%	/	/	

FH, feeding habits; H, herbivorous; C, carnivorous; O, omnivorous; Z, zooplanktivorous. The English names of fish species in the published studies are listed according to FishBase, a global information system on fishes.

$ English names are unavailable in FishBase, the English names in the published studies are presented;

# Specific Latin names are unavailable.

§ *Fish in the ponds were allowed to exist naturally without artificial feeding*.

Fish with controlled access to alternative diets have a changing GI microbial diversity and community structure (Ingerslev et al., 2014; Reveco et al., 2014) depending on digestive tract regions (Ye et al., 2014; Rhodes et al., 2016). Regardless of impacts of diets, fish exhibited significantly disparate clusters of microbial communities between stomach and intestine (Rhodes et al., 2016). Owing to the acidic gastric environment (Beasley et al., 2015), the stomach is viewed as a harsh territory resisting to exogenous microbial colonization, however, it is a place where chemical break-down of diets initiates. Despite the low gastric pH, microbial diversity in the stomach was still comparable to that in the intestine (Silva et al., 2011) and even higher in the stomach of fish (Xing et al., 2013). To some extent, a lot of the microbes are likely to be transient (Zhang C. et al., 2016), supporting the notion that the stomach acts as a sterilizing chamber for a bottleneck through which microbes can pass and passage into the intestine. Focusing on multiple terrestrial animals in recent studies, the microbial diversity in the stomach is not the lowest in the GI tract and feces (Keenan et al., 2013; Kohl et al., 2017). The main taxa in the stomach were similar to those in the intestine for some vertebrates (Bik et al., 2006; Keenan et al., 2013; von Rosenvinge et al., 2013). The distinct community patterns support divergent roles of the stomach and intestine in shaping microbial ecology. The taxonomic assignment of 16S rRNA sequences indicated that the stomach in vertebrates is dominated by *Firmicutes* largely contributing to microbial community differences between GI regions (Wu et al., 2012; Keenan et al., 2013). Like intestinal microbiota (Fetissov, 2017), gastric microbiota is involved in metabolism and homeostasis maintenance of the host. Significant differences in the metabolism between the stomach and intestine are suggested by the overrepresentation or underrepresentation of the predicted KEGG pathways associated with different metabolic processes and biosynthesis in the intestine or stomach. For example, in the stomach, we found higher levels of microbial functional genes associated with peptidases specializing in proteolysis into amino acids, while microbial functional genes involved in energy metabolism, amino acid metabolism, and lipid metabolism increased in the intestine. Preliminary food utilization in the stomach to a large extent depends on gastric acid production by amounts of host energy investment, and subsequent digestion and absorption in the intestine can rely on symbiotic microbiota for the provision of energy to the host. Microbiota needs to adapt to specific GI tract environment and then exerts effects on the host.

Food digestion by the GI tract is a dynamic, cyclical process. This accompanies changes in the microbial community for the utilization of substrates at different fermentative phases. When diet is replaced, gut microbiota can change rapidly within a day (David et al., 2014b). However, there were no consistent trends in microbial dynamics. This is largely due to fecal samples that are metabolic end products of original materials. They are representatives of a static status, as opposed to digesta within dynamic digestive processes. A key finding of the present study is temporal variations of microbial profiles observed after feeding, further indicative of the necessity to understand GI microbial ecosystems when analyzing microbiota in dynamic conditions.

We observed strong temporal fluctuations in relative abundance of phyla levels and lower taxonomic OTUs in this study, but not in alpha diversity, suggesting that short-term food digestion is sufficient to affect the taxonomic structure, less to the microbial members. The significant increase of *Bacteroidetes* after feeding is similar to that found in a 24h nutrient deprivation in mouse ceca (Crawford et al., 2009). As we see here, the abundance of *Bacteroidetes* is significantly elevated in the intestine of southern catfish at 24h after feeding, suggesting analogous trends of intestinal *Bacteroidetes* responding to diet availability in vertebrates. Nutrient shifts or deprivation lead to significant divergences in gut microbial ecology (Crawford et al., 2009) that might allow it to greatly benefit the host (Davis et al., 2016). Although dynamic transitions of fermentative chyme from the stomach into the intestine and pH changes were found after feeding, non-synchronization of changes occur in the microbial communities between stomach and intestine despite that they function together to digest food. The effects could be less pronounced when analyzing fecal microbiota. Therefore, we would be unable to reveal the scenarios of time-induced shifts during food digestion. Such substantial microbial variations in the digesta contribute to high individual-to-individual variations, and can be finally confused by confounding effects of host and environmental factors (Bolnick et al., 2014; Eichmiller et al., 2016).

## Conclusions

This study provides a more complete and dynamic picture regarding microbial community ecology in the GI tract of southern catfish. 16S rRNA gene-targeted sequencing showed differences between the stomach and intestine, indicating different microbial patterns across the GI tract. Using the initial diet factors (such as food types and food shifts), we are unable to explain the divergences of the GI tract microbiota. It is necessary to combine the digestive status with specific GI tract environment and digestive substrates to microbial variability. Therefore, we posit that the mechanisms underlying differences in microbial communities of GI tract may be correlated to dynamic ecological environments, such as host-accessible nutrients and diet transit time (or sampling time) as well as their interactions with microbial assemblages. Time-induced variations could be used to assess effect size to the differences within individuals or among studies. Moreover, it could be expected that, among other studies ignoring sampling time, similar problems should be obtained in the most common vertebrates that, like the results in this study, have dynamic rhythms of food digestion.

## Ethics statement

This study was carried out in accordance with the recommendations of Ethics Committee of Huazhong Agricultural University under approved permit number HZAUMO-2016-026.

## Author contributions

ZZ and DL designed the experiment. ZZ, WX, and MR conducted the experiment. ZZ analyzed the data. ZZ, DL, and MR wrote the manuscript.

### Conflict of interest statement

The authors declare that the research was conducted in the absence of any commercial or financial relationships that could be construed as a potential conflict of interest.
