# Supplementary material for: High Spatial and Temporal Variations of Microbial Community along the Southern Catfish Gastrointestinal Tract: Insights into Dynamic Food Digestion
Source: Front Microbiol. 2017 Aug 9;8:1531. doi: 10.3389/fmicb.2017.01531 (PMC5552716; doi:10.3389/fmicb.2017.01531)
Supplement: Supplementary file 1 [file Presentation1.PDF]

High spatial and temporal variations of microbial community along the southern catfish gastrointestinal tract: insights into dynamic food digestion

Zhimin Zhang [1, 2]**,** ***Dapeng Li*** [1, 2*]**,** ***Mohamed M. Refaey*** [1, 2, 3]**,** ***Weitong Xu*** [1, 2]

[1] *College of Fisheries, Huazhong Agricultural University, Wuhan, P.R. China,* [2] *Hubei Provincial Engineering Laboratory for Pond Aquaculture, Wuhan, P.R. China,* [3] *Department of Animal Production, Faculty of Agriculture, Mansoura University, Al-Mansoura, Egypt*

Running title: Gastrointestinal microbiota changes during food digestion

[*]Address correspondence to Dapeng Li, ldp@mail.hzau.edu.cn

Table S1. Body weight, body length and total length of southern catfish (*Silurus meriordinalis*) for gastrointestinal microbial analysis.

Groups	Body weight (g)	Body length (cm)	Total length (cm)
03 hour	493.4 ± 205.0	36.4 ± 5.5	40.6 ± 5.7
12 hour	455.9 ± 122.0	36.7 ± 3.3	40.7 ± 3.6
24 hour	480 ± 99.5	38.8 ± 2.7	42.2 ± 2.7

Table S2. Major microbial genera identified based on 16S rRNA results with mean relative abundance of more than 0.5% in any group at different time intervals at 03, 12, and 24h of gastrointestinal tract of southern catfish (*Silurus meriordinalis*) after feeding. Bold face denotes *P*-value < 0.05.

	Stomach			Intestine			
Genera	03h	12h	*P-value*	03h	12h	24h	*P-value*
Cetobacterium	44.48	14.69	***0.027***	65.51	61.06	44.02	***0.014***
Unclassified Clostridiaceae	23.03	35.76	***0.027***	1.5	2.47	7.84	***0.016***
Bacillus	7.68	6.58	0.208	1.19	0.03	0.04	0.216
Clostridium	7.08	26.55	***0.001***	0.92	1.19	2.58	0.153
Plesiomonas	4.74	5.64	0.674	18.56	20.82	8.51	***0.01***
Unclassified Bacteroidaceae	2.29	0.51	***0.002***	2.63	3.04	25.18	***0.007***
Unclassified Neisseriaceae	1.15	0.17	***0.003***	1.4	0.9	0.22	***0.003***
Unclassified Peptostreptococcaceae	1.08	0.61	0.141	2.1	0.58	2.91	***0.009***
Unclassified Oxalobacteraceae	0.65	0.18	0.834	0.01	0.03	0.01	0.141
Edwardsiella	0.55	2.7	***0.027***	1.69	5.52	3.3	0.054
Unclassified Enterobacteriaceae	0.54	0.38	0.6	0.04	0.02	0.01	***0.029***
Paucibacter	0.51	0.1	0.401	0.01	0.01	0	***0.033***
Porphyromonadaceae;Other	0.3	0.06	***0.002***	1.05	0.51	2.57	***0.013***
Unclassified Aeromonadaceae	0.25	1.32	0.059	0.04	0.74	0.02	***0.001***
Epulopiscium	0.21	0.07	0.6	0.69	0.08	0.55	***0.001***
Mycoplasma	0.17	0.13	0.172	0.83	1.81	0.72	***0.027***

Table S3. The contributions to dissimilarities between groups based on similarity percentage analysis (SIMPER) at phylum levels. Bold face in a column represents the two contributors (%) to overall dissimilarity between groups. The value in parentheses presents the overall dissimilarity between groups.

Taxon	Stomach vs Intestine (52.93%)	Int:03h vs Int:12h (13.87%)	Int:12h vs Int:24h (37.83%)	Int:03h vs Int:24h (36.23%)	Sto:03h vs Sto:12h (38.98%)	Int:03h vs Sto:12h (38.11%)	Int:12h vs Sto:24h (67.08%)
Firmicutes	***44.32***	17.26	14.07	12.76	***44.25***	***42.6***	***49.29***
Fusobacteria	***31.84***	***28.17***	***28.54***	***34.5***	***41.68***	***34.51***	***34.23***
Proteobacteria	11.91	***36.84***	21.46	16.87	9.805	17.19	12.48
Bacteroidetes	10.18	12.84	***33.86***	***34.66***	3.005	3.628	2.125
Tenericutes	0.9103	4.394	1.745	0.9018	0.09951	0.8625	1.247
Actinobacteria	0.2848	0.1141	0.03362	0.02476	0.3419	0.4023	0.2098
Unassigned;Other	0.2459	0.1743	0.1896	0.1756	0.3809	0.3194	0.2293
[Thermi]	0.1372	0.05603	0.02148	0.00897	0.1459	0.2127	0.08995
Nitrospirae	0.03118	0.009164	0.002615	0.00312	0.06954	0.07534	0.006292
Chlorobi	0.02983	0.07387	0.06317	0.0759	0.03602	0.03712	0.02241
Verrucomicrobia	0.02719	0.002036	0.0007467	0.001364	0.05843	0.062	0.007826
Acidobacteria	0.01835	0	0.001497	0.001563	0.03109	0.03346	0.01024
Chloroflexi	0.01644	0.04534	0.007659	0.01697	0.02284	0.01819	0.01669
Cyanobacteria	0.01298	0.004073	0.001493	0	0.02026	0.01487	0.0119
Lentisphaerae	0.007335	0	0	0	0.01664	0.004478	0.009032
Armatimonadetes	0.006824	0.002036	0.002615	0.002341	0.008014	0.01264	0.004009
Planctomycetes	0.00621	0.002036	0.001307	0.0007797	0.01232	0.006683	0.006109
GN02	0.002494	0	0.0007467	0.0007797	0.005989	0.00594	0.0004211
Crenarchaeota	0.001222	0	0	0	0.003037	0.002239	0.0006569
[Caldithrix]	0.001062	0	0.0007484	0.0007814	0.002219	0.001484	0.0006569
OP3	0.0003562	0.002036	0.001308	0.0007814	0	0	0.0004211

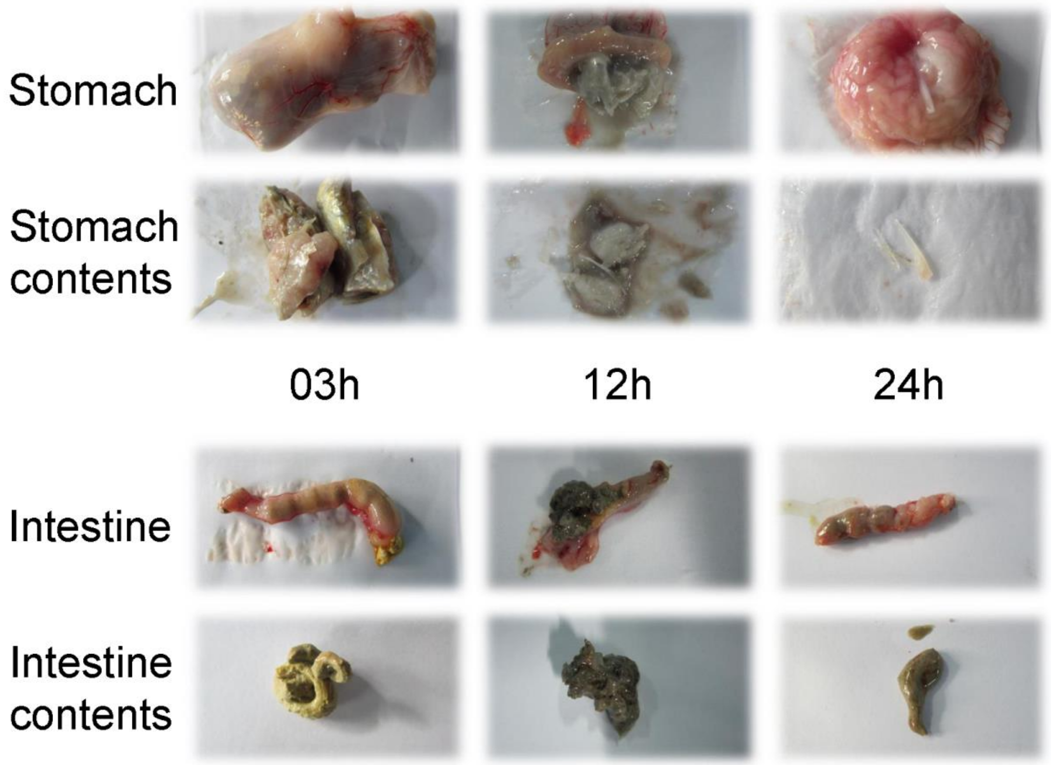

Figure S1. The GI tract and their contents of southern catfish (*Silurus meriordinalis*) at different time intervals at 03, 12, and 24h after feeding.

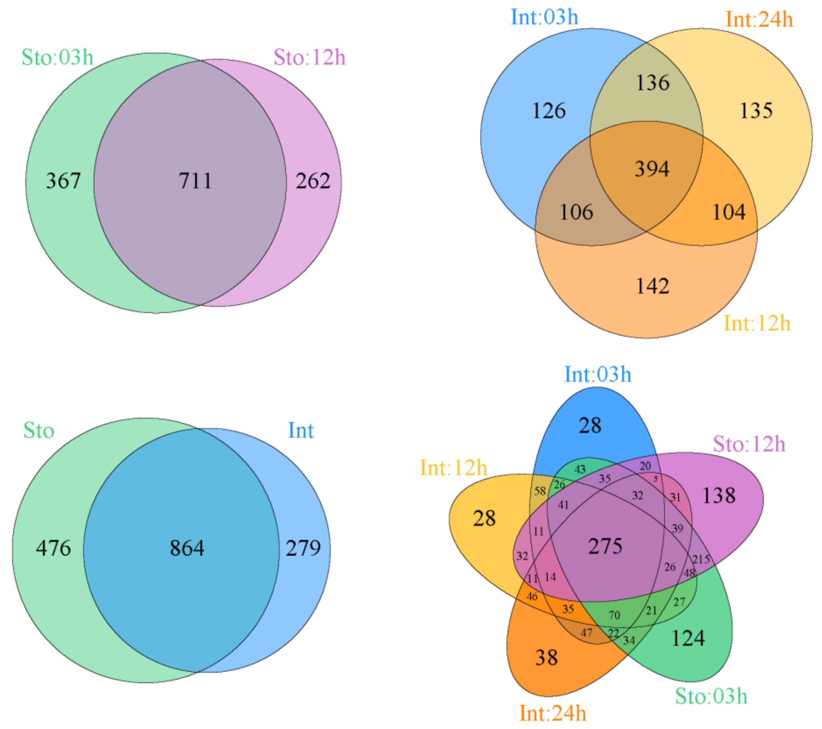

Figure S2. Venn diagram showing the unique and shared OTUs of GI tract of southern catfish (*Silurus meriordinalis*). Sto, stomach; Int, intestine.

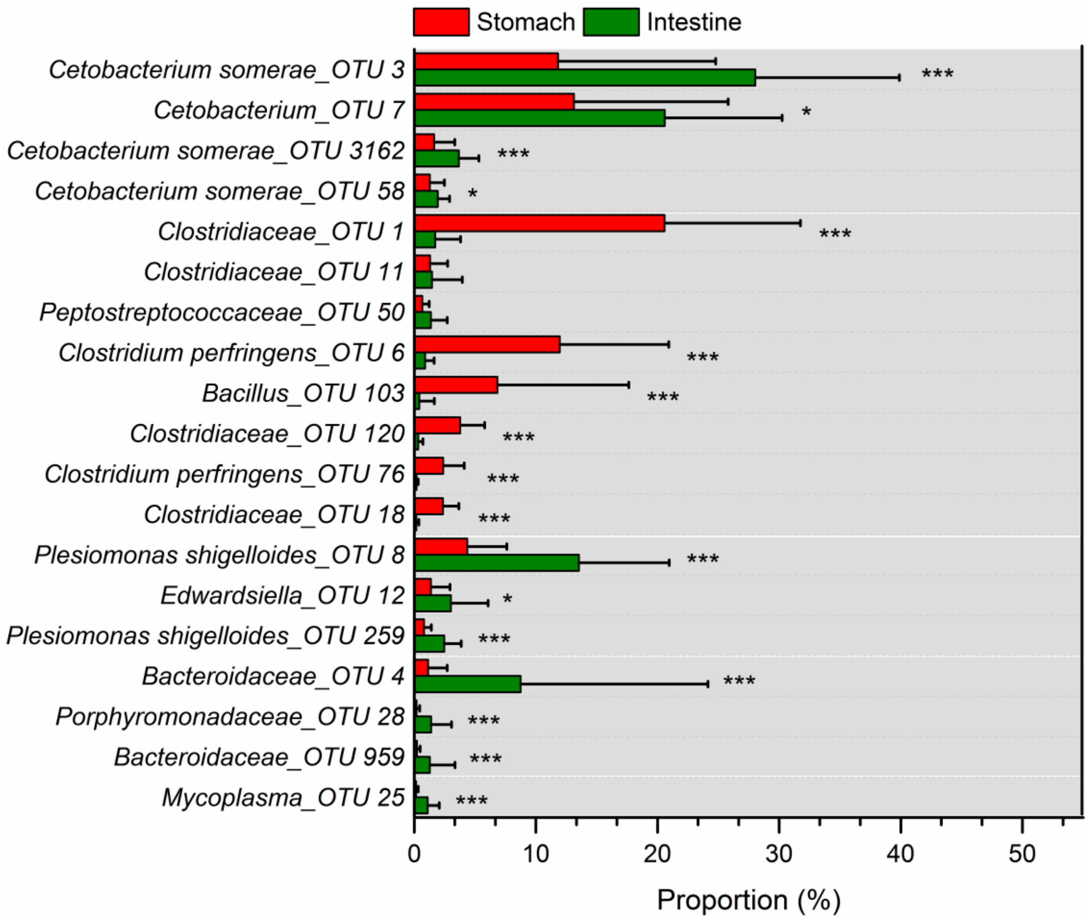

Figure S3. Comparisons of the taxonomic compositions in the gastrointestinal tract microbiota of southern catfish. The relative abundances (> 0.5%) for the top 19 taxa at the OTU levels between stomach and intestine are presented. Asterisks indicate significant differences (**P* < 0.05, ***P* < 0.01, ****P* < 0.001).

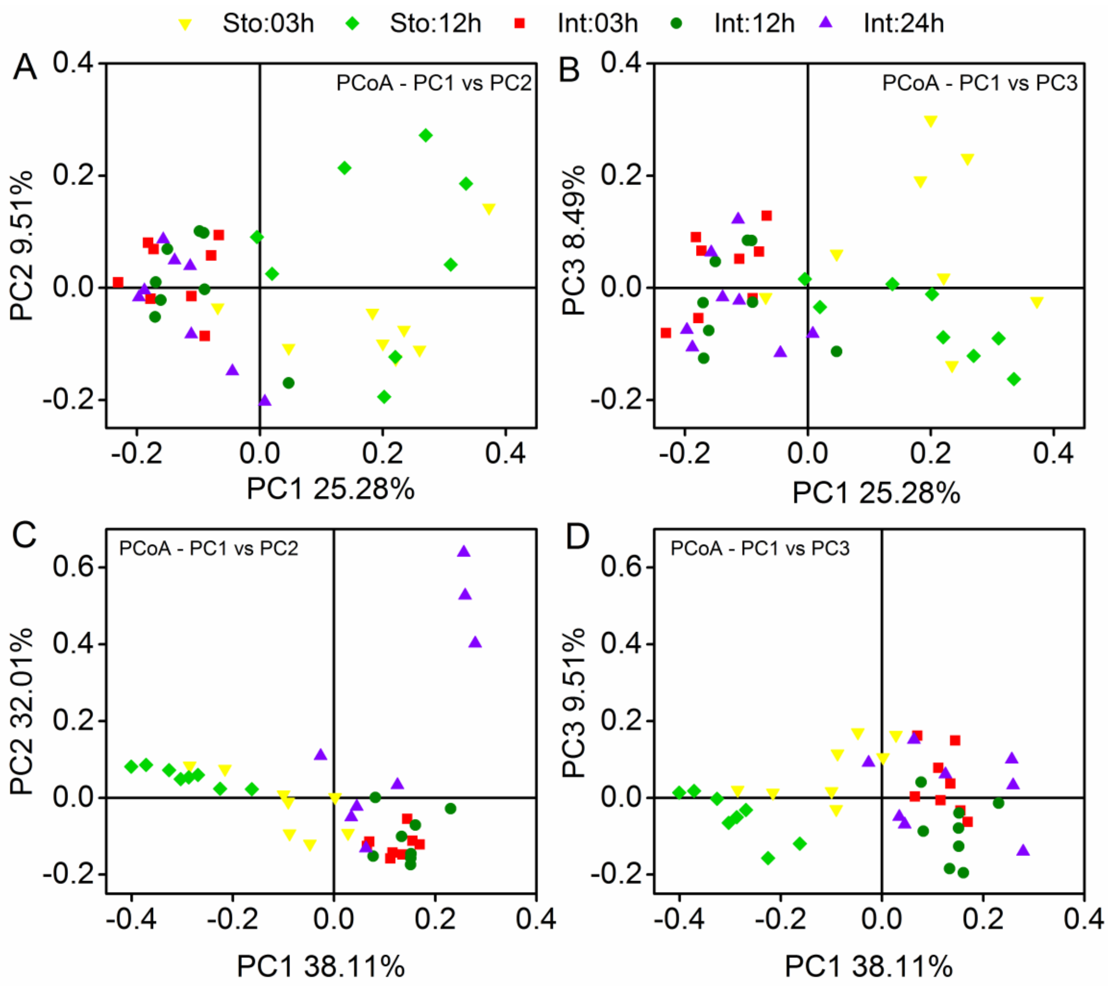

Figure S4. Score plots of principal coordinate analysis of gastrointestinal microbiota based on unweighted (A and B) and weighted (C and D) UniFrac distances.

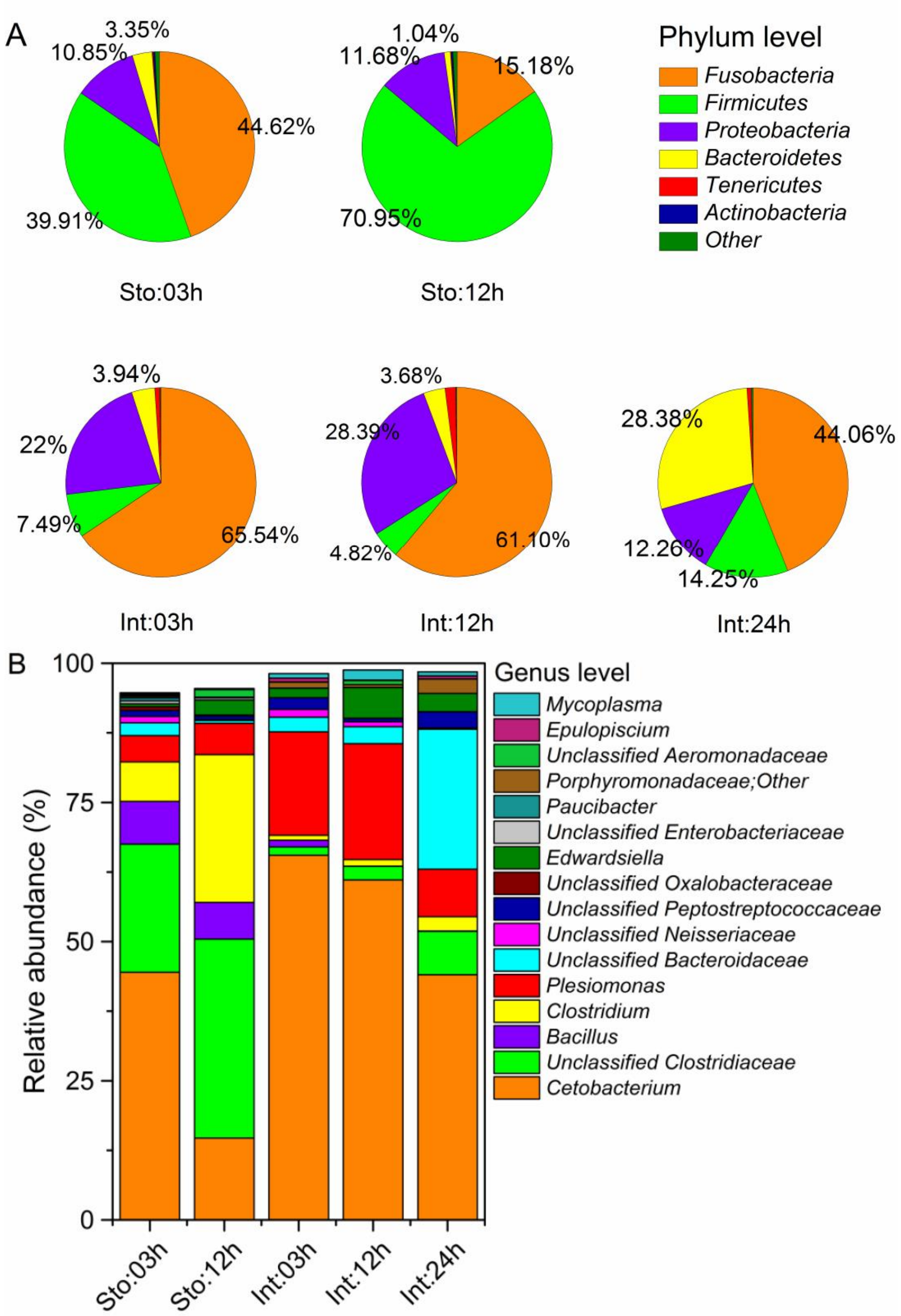

Figure S5. Relative abundance of the dominant microbial compositions in the gastrointestinal tract of southern catfish at different time points after feeding. The relative abundance (A) at the phylum level, and (B) at the genus level.

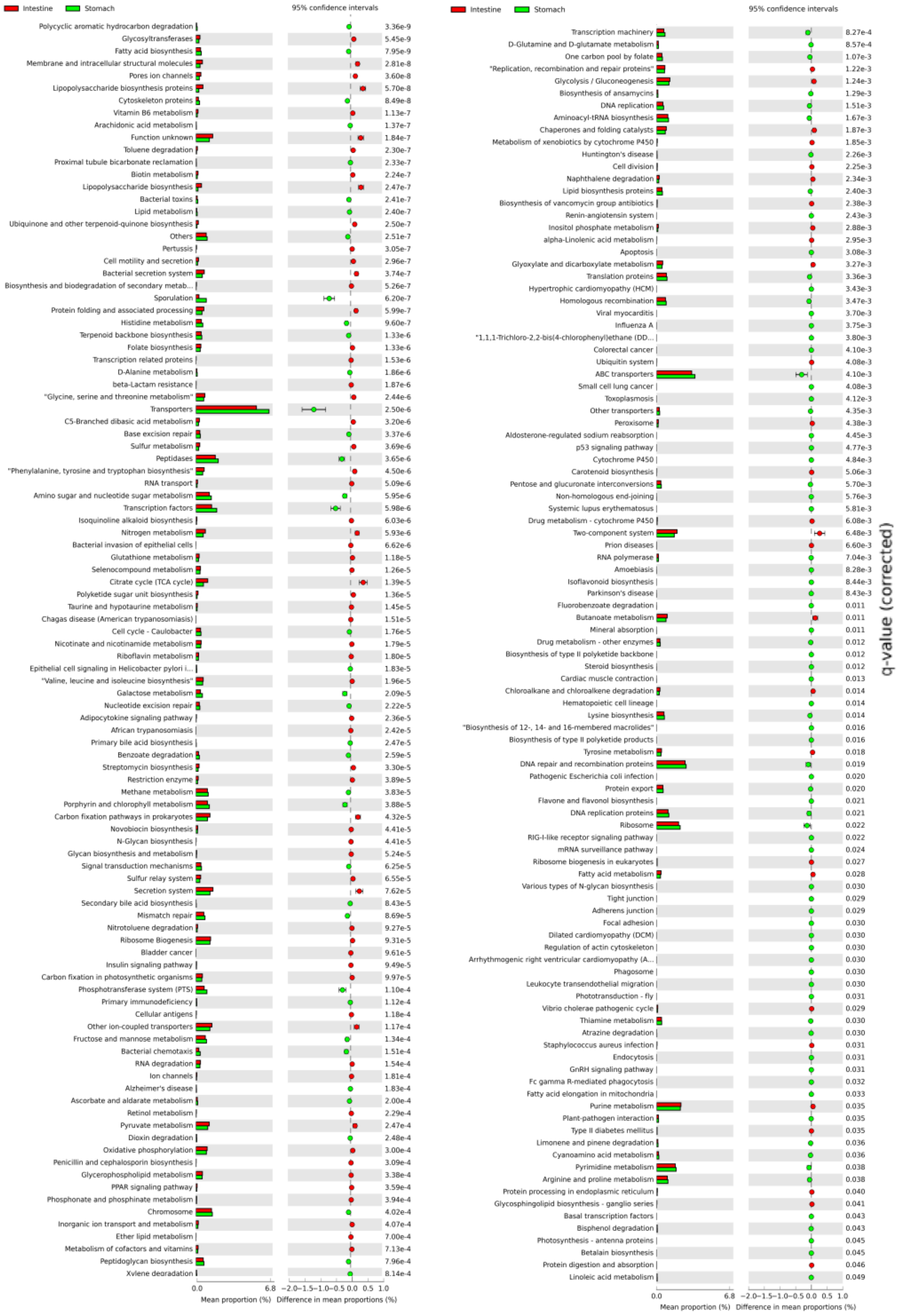

Figure S6. Mean proportions and the differences in predicted functional genes of the gastrointestinal tract microbiota at KEGG level 3.

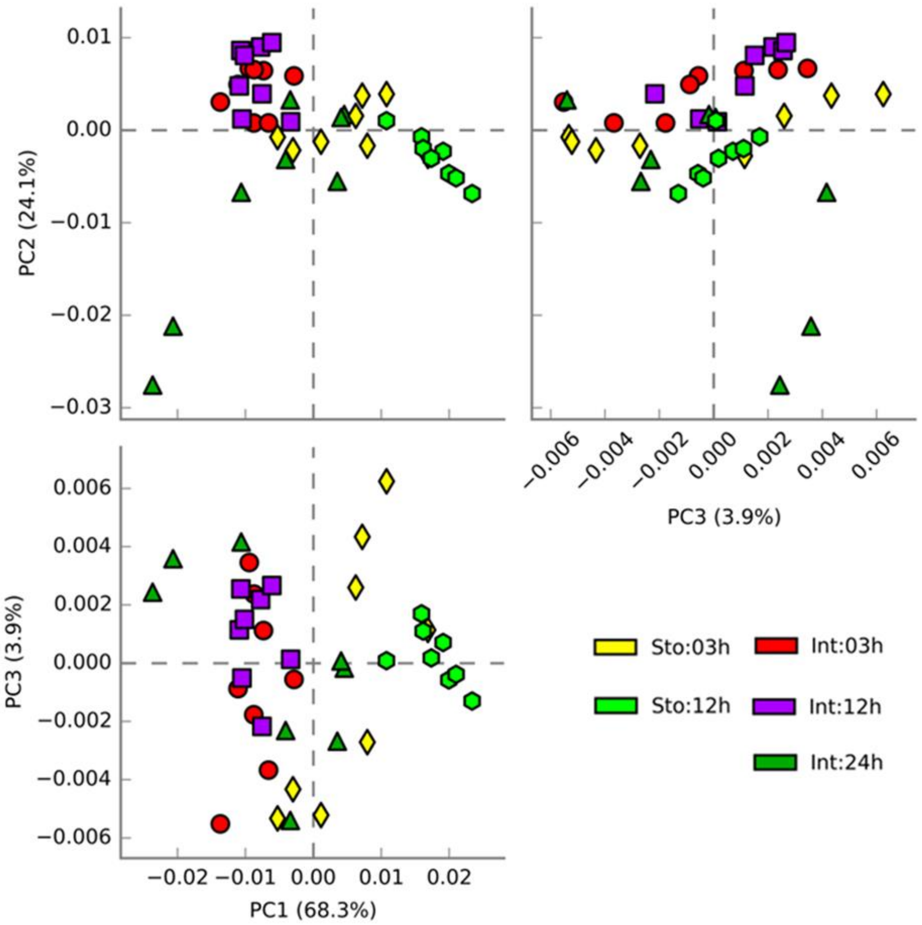

Figure S7. Principal components analysis reveals temporal differences of predicted functional genes at KEGG level 3 associated with microbiota in the gastrointestinal tract during the digestion.
